# The Effect of Selected Operational Factors on the Vibroactivity of Upper Gearbox Housings Made of Composite Materials

**DOI:** 10.3390/s19194240

**Published:** 2019-09-29

**Authors:** Tomasz Figlus, Mateusz Kozioł, Łukasz Kuczyński

**Affiliations:** 1 Faculty of Transport, Silesian University of Technology, 8 Krasinskiego Street, 40-019 Katowice, Poland; luk.kuczynski@gmail.com; 2 Faculty of Materials Engineering and Metallurgy, Silesian University of Technology, 8 Krasinskiego Street, 40-019 Katowice, Poland; mateusz.koziol@polsl.pl

**Keywords:** vibration, noise, gearbox, fiber reinforced polymer composite

## Abstract

This paper presents the results of laboratory tests in which evaluation was performed regarding the effect of selected operating factors on the vibroactivity of upper gearbox housings made of three different fiber reinforced polymer composite materials with diverse layouts (cross and random) and types of reinforcing fibers: glass fiber and carbon fiber. The results of tests for composite housings were compared with those for a steel housing. The tests showed that composite housings had a weight lower by more than 60% compared to the steel housing. A multisensor measuring system consisting of vibration acceleration transducers, a directional microphone and a data acquisition card with software was used for the study. Tests of the vibroactivity of upper gear housings were carried out at different loads and rotational speeds of toothed gears. The study showed that composite housings are less sensitive to changes in the rotational speed that steel housings. The tests showed that at a higher rotational speed of the gear transmission, housings made of composite materials had a comparable or lower level of vibration. Tests and analyses of the vibroactivity of housings performed at different loads of the gear allow the conclusion that composite housings, despite a considerably lower weight than steel housings, are less sensitive to changes in the load of the gearing.

## 1. Introduction

One of the sources of vibration and noise of means of transport is the power transmission system, where the gear constitutes the main source of vibroacoustic energy [1,2,3,4,5,6,7,8,9]. The energy level emitted by toothed gears depends on structural, technological and operational factors. The main source of gear vibration is the process of teeth meshing, the bearings and the occurrence of clearance, as well as wear or damage to the components. Occurring vibrations are transmitted to the housing and farther, to the environment, as noise.

Several generally acceptable methods of minimizing the vibroacoustic activity of gears [1,5,10,11] have emerged from the research conducted both in laboratory and industrial conditions, and with the use of computer simulation:

- The application of a tooth profile relevant for the gear load in order to reduce dynamic effects, thereby reducing also the vibration and noise of the gearbox [3,10,12].
- A change of gear parameters, making wheels in higher accuracy classes, the use of bevel teeth instead of straight ones, the use of high teeth [13,14,15,16,17].
- The use of favorable bearing methods to dampen the dynamic actions transferred from shafts to bearing housings (use of slide bearings instead of rolling bearings) [18,19,20,21,22].
- Shaping of the gear housing in order to minimize vibration at a preset forced gearing [1,23,24,25].
- The use of damping inserts inside the toothed wheel disk or in the housing under the bearings [1,3,7,10].
- The use, between the toothed gear and the shaft, of a multilayer vibration-damping sleeve [1,3,7,10].
- The application of the active vibration reduction method [1].

In addition, as results from other studies have shown [1,3,26,27,28,29,30,31,32,33,34], the main source of noise generated by the gear transmission is its housing and, in particular, its upper lid. Modification of the upper housing by introducing additional ribs significantly contributed to a reduction of its vibroactivity [1,29]. Another solution that is recommended to minimize the vibroactivity of the gear housing is to modify its wall thickness [28,32]. Increasing the wall thickness leads to a significant reduction in the vibroactivity of the gearbox; however, at the expense of increasing the weight of the housing.

The arrangement of ribs and their geometric properties (width, height) also have a significant influence on the vibroactivity of the gear transmission housing. As studies have shown [29], an advantageous solution is to adopt a cross-section of the ribbing in the proportions: height/thickness = 4 and width/thickness = 1. In this case, a reduction of the vibroactivity of more than 4.5 dB is achieved, with a less than 5% increase in the weight of the housing compared to the unribbed housing.

When constructing the gearbox housing, solutions should be avoided where the structure would strengthen the vibration coming from of interacting elements, e.g., the meshing frequency or their harmonics. These places play the role of an acoustic resonator, thus, they are a source of high intensity noise.

Various methods of measuring vibroacoustic signals are used to study the vibrations and noise of means of transport, including toothed gears [1,2,4,29]. The most often used measurement method is the use of piezoelectric transducers [34,35,36,37,38,39,40], which record the acceleration of vibration at selected measurement points. Measurement of this type is a contact method, as it requires attaching a transducer, which may be difficult or sometimes impossible. In studies of vibration, techniques of contactless measurement of vibroacoustic signals by means of laser vibrometers can be applied [41]. Such a measurement method is gaining interest in studies requiring measurements to be taken at places difficult to access, from a considerable distance or at high temperatures. Other methods of signal measurement used in vibroacoustic tests include the use of acoustic measurements by means of directional microphones or, for example, acoustic holography [20,34]. These test methods are currently being developed, as they enable, first of all, contactless measurements.

Reducing the weight of single components within the means of transport, while maintaining the required mechanical strength, also reduces energy consumption by these means of transport. One way to reduce the weight is to use lightweight construction materials of high strength. These requirements are met by composite materials based on metal and polymer alloys, which are increasingly used in mechanical engineering and transport [42,43,44,45,46,47,48,49,50,51].

A very important factor influencing the magnitude of noise and vibration generated by the gearbox housing and its mass is the material of which the housing is made. The most popular materials for this type of elements are steel or cast iron. This is due to both technical reasons—they are durable materials—and economic reasons—the steel and cast iron technology is well-developed and the unit price of the product is low in the case of mass production. However, steels and cast iron are not ideal materials. In the case of low-volume or unit production (e.g., reproduction of an element according to an adaptation design), the manufacturing costs are very high. These materials may also be exposed to catastrophic cracking in the case of unfavorable and accidentally obtained significant dynamic loads [52,53]. Typical (cheap) steels and cast iron have a weak specific strength compared to other engineering materials [54], i.e., products made of them are heavy. Modern alternatives to steel or cast iron for gear housings are fiber reinforced plastic (FRP) composites, including, first of all, glass fiber reinforced plastic (GFRP) and carbon fiber reinforced plastic (CFRP)—the latter type for more advanced and demanding construction cases. FRP composites are materials composed of two basic phases: high-strength reinforcement in the form of fibers and a relatively soft, viscoelastic polymer matrix. These two phases differ fundamentally, including in terms of the modulus of elasticity (a difference of circa 50–200 times [55,56]), which is of fundamental importance for the conduction and damping of elastic waves. Due to the large difference in the modulus of elasticity between the two components, the presence of a phase boundary becomes very important, and its specific surface is very large in the FRP type composites. This has a fundamental influence on the conduction of elastic waves and their damping by multiple scattering [57]. The damping takes place mainly at high frequencies and concentrations [58,59]. The results obtained by the authors, inter alia, in their earlier works, [60] and [61], prove that FRP composites are suitable as a vibroacoustic material. 

At the same time, however, FRP composites as construction materials for gear housings have not been sufficiently tested thus far. They have not been used on a large scale, but are known to be effectively applied in the gearbox housings of Formula 1 cars. Compared to cast iron or steel, FRP composites are much lighter (at least by 60% [59]). Their mechanical properties [62] are undoubtedly sufficient enough to meet the design requirements of gear housings. Additionally, their thermal resistance allows them to be used in this range (they can operate at temperatures up to 120 °C). FRP composites show sufficient rheological strength (creep, stress relaxation) and fatigue strength. A significant advantage of these materials is their high energy absorption capacity [63,64], which results from the complex structure and viscoelastic properties of the polymeric matrix. FRP composites are designable materials and their mechanical properties depend mainly on the content and direction of reinforcing fibers [56].

In recent years, the knowledge about the properties of FRP composites, which may be important for their use in gearbox housings, has significantly increased. It has been found, inter alia, that these composites have relatively high resistance to liquids absorption at elevated temperatures [65] and very good impact resistance [63]. The internal stress distribution in FRP composites is quite complicated and is based on the transfer of local loads through the matrix to the fibers [56]. This internal behavior of FRP composite, which takes place over the entire, very large specific surface of phase boundaries, causes in an even distribution of stresses inside the material, which helps to increase its strength. An example of a technical solution that takes advantage of the uniform distribution of working stresses of FRP composites is the integrated piezoelectric sensor described in study [66]. 

This study addresses the problem of assessment of the vibroactivity of gearbox housings made of three selected composite materials: a GFRP composite with a cross layout of fibers (plain woven glass fabric reinforcement), a GFRP composite with a random layout (glass chopped strand mat reinforcement) and a CFRP composite with a cross layout (carbon twill weave fabric reinforcement). The obtained results were compared to the results recorded for the solution of a classical housing made by welding of steel sheets and sections. The analyzed composite housing solutions were characterized by a lower mass by at least 60% compared to the steel housing, while maintaining their geometric similarity. The research was focused on the evaluation of vibroactivity during experiments at a laboratory stand in variable load conditions and speed of the gear. 

The study presented in the publication is a continuation of the research presented in [59], where it was analyzed how the mass and vibroactivity changed (during modal analysis studies) for gearbox housings made of three different composite materials. 

## 2. Materials and Methods

For vibroactivity testing, housings made of steel, marked as Steel Housing, and of three different composite materials, marked as Housing K_1–K_3, were used. The housings were:

1. Steel Housing—a housing made of steel sheets and sections, with steel inserts of bearing nodes, made of the St3 steel by welding. Weight—13 kg.
2. K_1—a housing made of a GFRP composite reinforced with plain woven glass fabric, areal mass 300 g/m^2^ (produced by KROSGLASS, Krosno, Poland), 24 layers; matrix: chemically-catalyzed polyester resin ESTROMAL 14 LM (produced by LERG, Pustkow, Poland); formed by hand lay-up method with vacuum assistance [67]; steel inserts of bearing nodes bonded to the composite housing with high endurance epoxy adhesive. Weight—4.4 kg (66.2% weight reduction in relation to the Steel Housing).
3. K_2—a housing made of a GFRP composite reinforced with chopped strand mat, areal mass 540 g/m^2^ (produced by KROSGLASS, Krosno, Poland), nine layers; matrix: chemically-catalyzed polyester resin ESTROMAL 14 LM (produced by LERG, Pustkow, Poland); formed by hand lay-up method with vacuum assistance [67]; steel inserts of bearing nodes bonded to the composite housing with high endurance epoxy adhesive. Weight—5.2 kg (60 % weight reduction in relation to the Steel Housing).
4. K_3—a housing made of a CFRP composite formed in autoclave; the input material were prepregs of twill weave carbon fabric (2 × 2) with a basis weight of 800 g/m^2^ (six internal construction layers) and 240 g/m^2^ (external visual layers); the matrix was epoxy resin in the precured state contained in the prepregs; the prepregs were produced by DELTA PREG, Sant’Egidio alla Vibrata, Italy; pressure difference during the process: 5 bar, basic temperature of the process: 120 °C, process time 4 h; steel inserts of bearing nodes bonded to the composite housing with high endurance epoxy adhesive. Weight—4.7 kg (63.8% weight reduction in relation to the Steel Housing).

**Figure 1:**
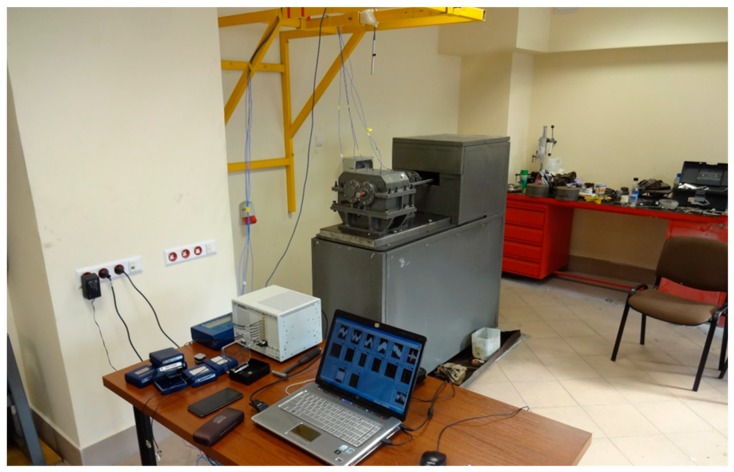
View of the FZG stand and the measuring system for stand tests [59].

**Figure 2:**
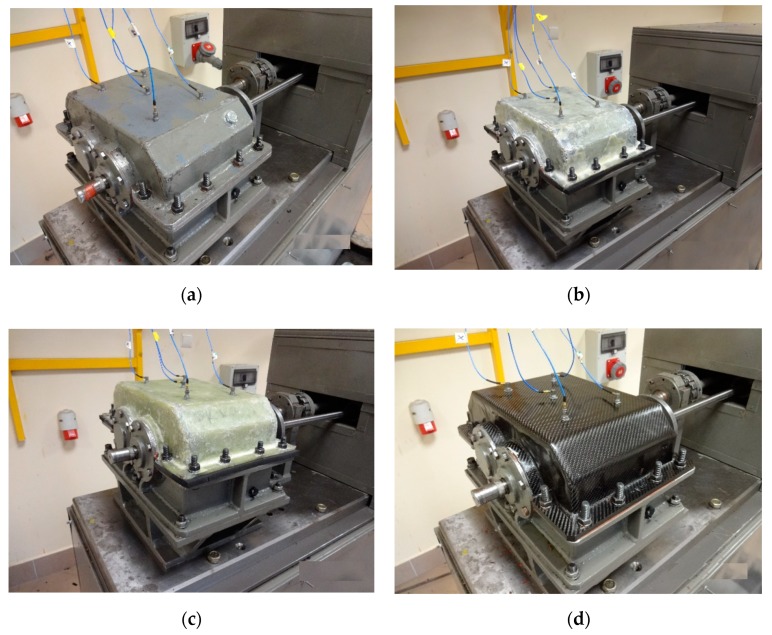
View of housings mounted on the stand together with measuring transducers [59]: (**a**) Steel housing; (**b**) K_1 housing; (**c**) K_2 housing; (**d**) K_3 housing.

The shape of the housings corresponded to that of the steel housing in accordance with geometric similarity assumptions (Thickness of the wall = 6 mm). Detailed information on the materials of which the housings were made and the manufacturing technology is included in papers [59]. Figure 1 and Figure 2 present upper gearbox housings intended for tests. 

The tests were performed in the Laboratory for Power Transmission Systems at the Faculty of Transport, Silesian University of Technology. The measuring stand and distribution of measurement points are described in detail in the Authors’ paper [59].

The tests were carried out using a measurement system, which consisted of (Figure 1 and Figure 2):

- Five vibration acceleration transducers—vibration measurement of the upper plate of the housing—point P_1–P_5.
- Directional microphone—noise measurement at a distance of 0.5 m above the upper plate of the housing—point H.
- National Instrument NI 4472 data acquisition card—processing and recording of signals.
- LabView software—measurement control and signal recording.
- Matlab software—signal processing.
  Sampling frequency of 20 kHz was adopted during simultaneous signal recording.

**Figure 3:**
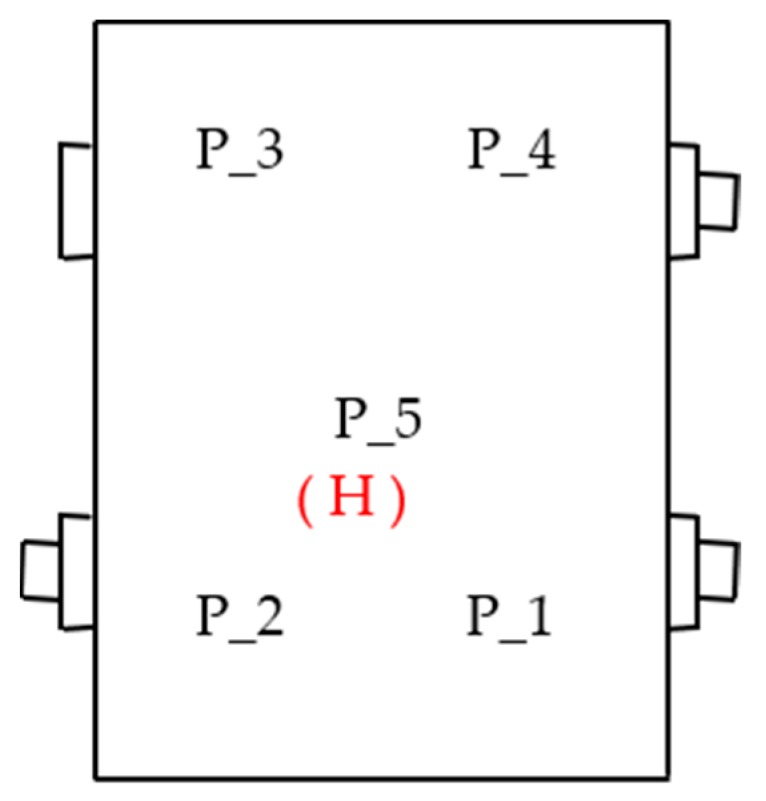
Arrangement of the measurement points of vibration (P) and noise (H).

Figure 3 shows the arrangement of the points of induction and measurement of vibration and noise.

## 3. Results and Discussion

### 3.1. Identification of the Resonant Structure of Gearbox Housings and Their Vibroactivity Using Start-up Characteristics

The application of start-up and coasting characteristics in the research allows identifying the resonant structure of the gearbox housing and its vibroactivity, depending mainly on the geometric features of the gearing and the rotational speed of the shafts. This approach enables the stimulation of housing vibration by real induction generated from the gearing through shafts and bearing nodes, to the bearing hubs of the housing. In the case of gear mesh, such a signal has numerous constituents related to the rotational frequencies of the pinion and wheel, the gear mesh frequencies, including sidebands, and their harmonics. As can be seen from the studies presented in [1,29,34], these frequencies are characterized by considerable energy, which affects significant excitation of housing vibrations and increases the noise level of the gear. 

It was assumed that the Steel housing and composite housings K_1–K_3 would be subjected to tests identifying resonance frequencies and vibroactivity by means of start-up characteristics. When planning the experiments, it was assumed that the test stand would be equipped with bevel-toothed wheels, which, as part of the studies presented in [34], were characterized by a low level of vibroactivity occurring within a wide range of rotational speed. Geometrical parameters of the wheels are compiled in Table 1. 

During the measurements, it was also assumed that the rotational speed of the shaft would change in a linear way within the speed range of 0–2400 rpm. The unit load at the gear meshing point during this experiment was *Q* = 1.5 MPa. 

On the basis of the recorded vibration acceleration and noise signals at a distance of 0.5 m above the gearbox housing, their time and frequency characteristics were determined using a fast Fourier transform (marked as *fft*). The calculations were made in the time window of 0.125 s in the Matlab program, on the basis of the dependence:

(1) $$Y\left(k\right)=\sum_{j=1}^{n}X\left(j\right)W_{n}^{\left(j-1\right)\left(k-1\right)}$$

where: $W_{n}=e^{\left(-2\pi i\right)/n}$ and *n*—signal length.

**Figure 4:**
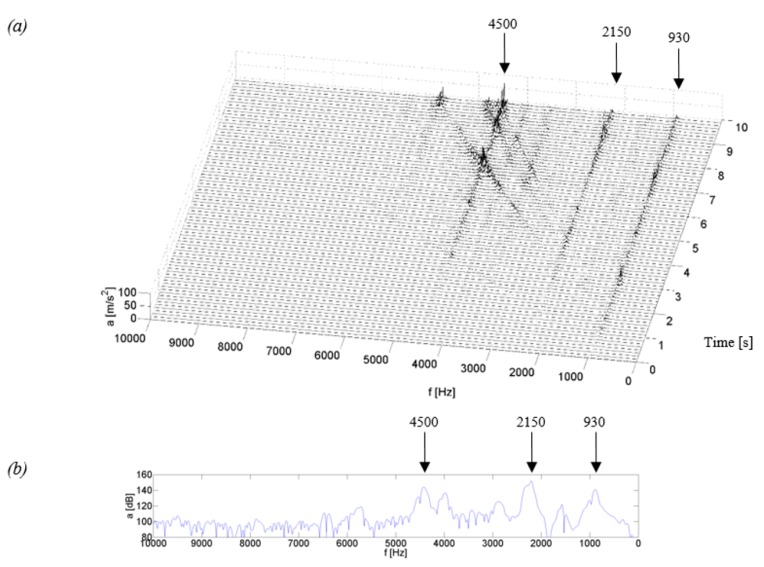

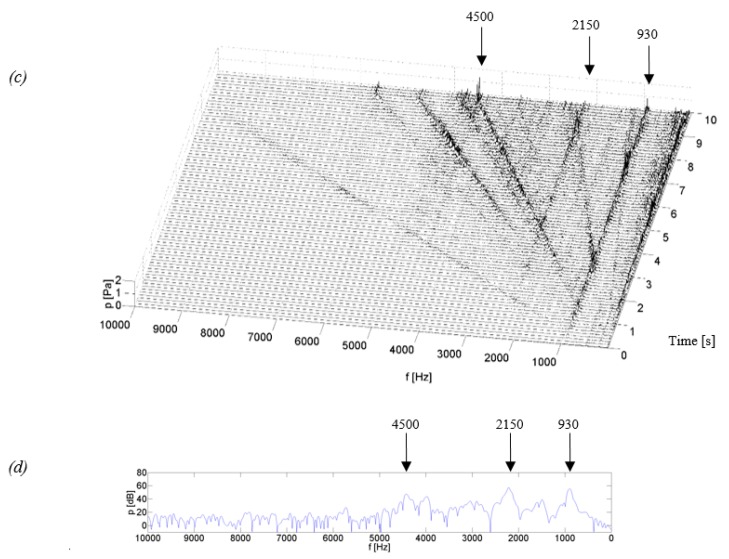
Test results for the Steel housing: (**a**) time-frequency distributions of vibration acceleration (P_5), (**b**) vibration response (P_5), (**c**) time-frequency distributions of acoustic pressure (*H*), (**d**) acoustic response (*H*).

**Figure 5:**
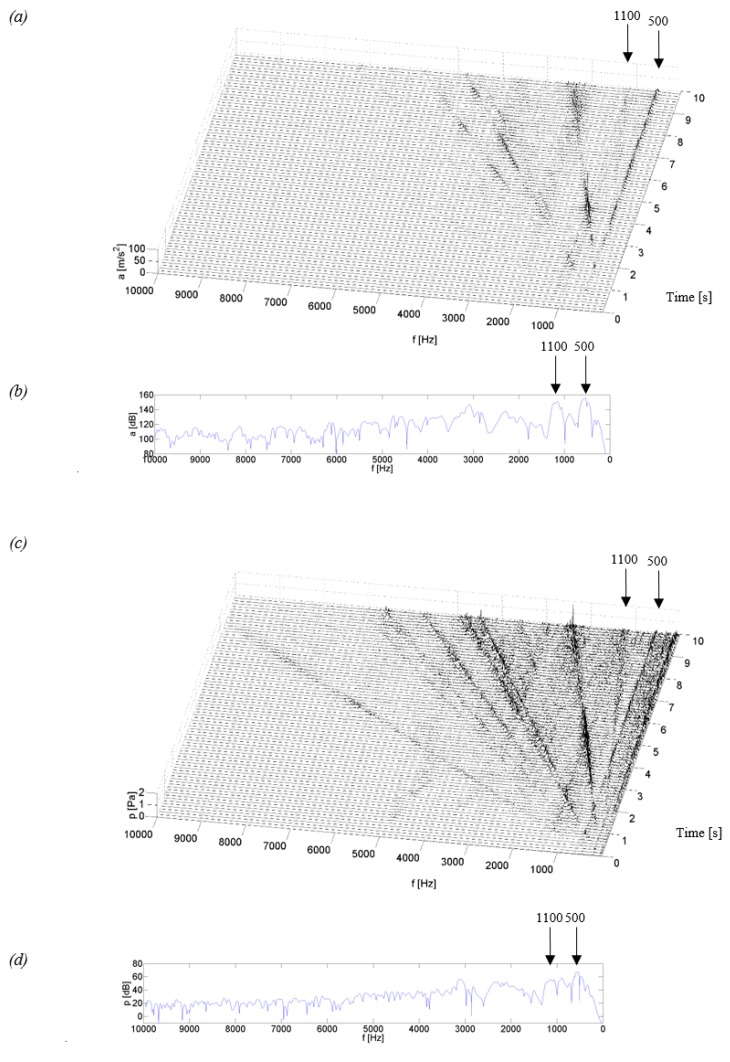
Test results for the K_1 housing: (**a**) time-frequency distributions of vibration acceleration (P_5); (**b**) vibration response (P_5); (**c**) time-frequency distributions of acoustic pressure (*H*); (**d**) acoustic response (*H*).

**Figure 6:**
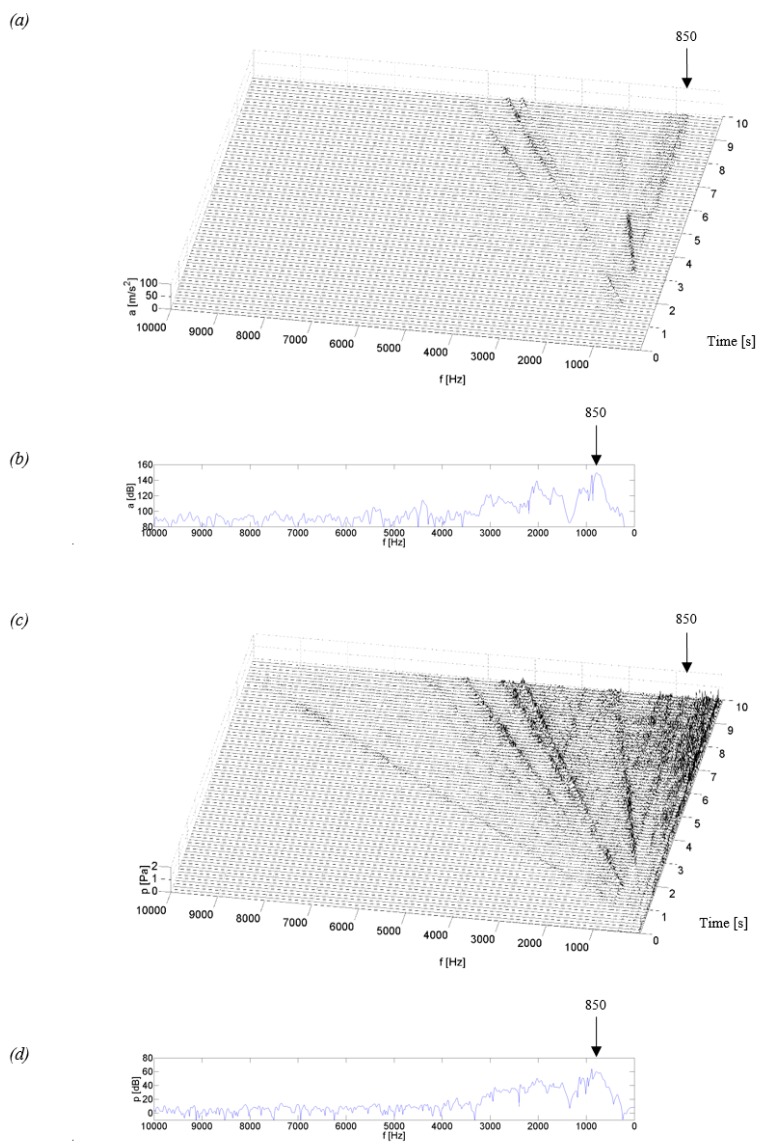
Test results for the K_2 housing: (**a**) time-frequency distributions of vibration acceleration (P_5), (**b**) vibration response (P_5), (**c**) time-frequency distributions of acoustic pressure (*H*), (**d**) acoustic response (*H*).

**Figure 7:**
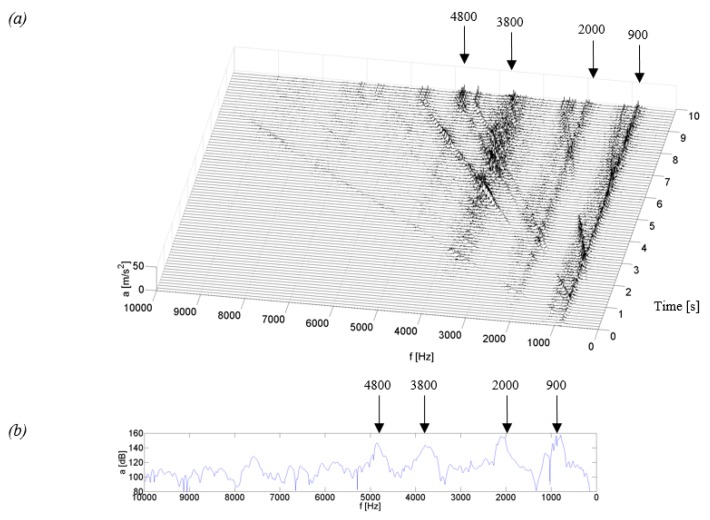

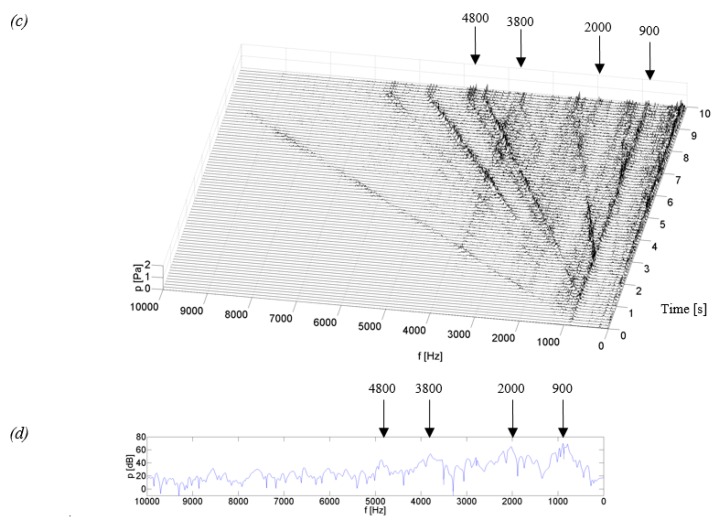
Test results for the K_3 housing: (**a**) time-frequency distributions of vibration acceleration (P_5), (**b**) vibration response (P_5), (**c**) time-frequency distributions of acoustic pressure (*H*), (**d**) acoustic response (*H*).

Figure 4, Figure 5, Figure 6 and Figure 7 show examples of time-frequency distributions of vibration acceleration and noise signals, together with the resonance characteristics of the measuring point determined during the modal analysis of housings mounted on the stand and discussed in paper [59]. Analysis of the results of the upper gearbox resonance response tests, presented in [59], showed that the K_1–K_3 composite housings in a lower frequency range—below 1 kHz—are characterized by the occurrence of resonant frequencies with a higher amplitude than the steel housing. At higher frequency ranges—above 1 kHz—the composite housings had a lower vibroactivity level than the steel housing.

It results from these investigations that the vibration and acoustic response of the steel housing and composite housings K_1–K_3 is different. 

In the time-frequency distributions of signals generated by the steel housing (Figure 4), three main resonance frequencies can be observed at 0.93, 2.15 and 4.5 kHz, as well as three resonance frequencies of lower energy, occurring at frequencies of circa 1.5, 3.5 and 4.0 kHz. In the distributions, a local, linear increase in signal amplitude can be observed, which is related to the increasing rotational speed of the gear and is caused by characteristic gear mesh frequencies along with sidebands and their harmonics. These frequencies, with a change of rotational speed, meet with the resonance frequencies of the housing, thereby causing local increases in the signal amplitude, which can be observed, among others, during the measurement time of approximately 3.2, 7.1 or 9 s. This results in the situation where in the signals of vibration acceleration and acoustic pressure of the Steel housing, an increase is observed in the amplitude, also at higher rotational speeds. This is particularly noticeable when the characteristic frequencies of the gear meshing intersect the resonance frequency of circa 4.5 kHz, where a significant vibration excitation occurs at the measuring point.

Comparing the resonant frequencies which occur in the time-frequency distributions in Figure 4a,c, and those determined in the modal analysis in Figure 4b,d, it can be concluded that the frequency values of 0.93, 2.15 and 4.5 kHz are convergent in both methods of measurement, while the frequencies with lower energy of circa 1.5 and 3.5 kHz were excited only during the normal operation of the stand. Analyzing the signal amplitude at a frequency of circa 4.0 kHz, it can be concluded that during the tests using modal analysis, this frequency showed a significant resonance of the housing, which was no longer confirmed during the coasting test and the amplitude of signals at this frequency had a much lower value.

Analyzing the test results obtained for the K_1 and K_2 housings made of glass fiber reinforced composite (fabric or mat) with a polyester resin matrix (Figure 5 and Figure 6), a similar vibroacoustic response can be observed. In the time-frequency distributions, local resonance of the gearbox housing is observed, of a higher value for the K_1 housing at frequencies of circa 0.5 and 1.1 kHz, and of a lower value of circa 2.1 and 3.2 kHz. For the K_2 housing, the resonance of a higher value is observed only at a frequency of circa 0.85 kHz, while the remaining increases in the signal amplitude had much lower, insignificant values. The results of these tests indicate that the composite housings K_1 and K_2 are characterized by significant vibroactivity in the low range of rotational speed, where characteristic rotational frequencies of the meshing and their harmonics meet with the resonant frequencies of the housings. As the rotational speed of the gear increases (measurement time after 5.5 s), a significant local increase in the signal amplitude caused by the passage of characteristic frequencies through resonance is no longer observed in the time-frequency distributions. This indicates a lower vibration activity of the K_1 and K_2 housings at a higher gear speed. 

Time-frequency distributions of vibration signals and acoustic pressure—Figure 5a,c and Figure 6a,c—have confirmed the modal analysis results for the selected measuring point—Figure 5b,d and Figure 6b,d—where the occurrence of resonant frequencies is also observed in the lower range of frequencies.

Analyzing the test results obtained for the K_1 and K_2 housings and the Steel housing, it can be concluded that the composite housings are distinguished by higher vibroactivity in the range of rotational speed lower than in the case of the steel housing. At a higher rotational speed, the composite housings showed a lower vibroactivity than the steel housing. This is caused by the occurrence of resonance frequencies for the steel housing also in higher ranges of the analyzed band, which is not the case with composite housings K_1 and K_2.

Identification of the resonant structure and vibroactivity during the coasting test of the K_3 housing made of a carbon fiber reinforced composite showed that the resonance of the housing occurred at frequencies of approximately 0.9, 2.0, 3.8 and 4.8 kHz (Figure 7). In the time-frequency distributions, lines of signal amplitude growth can be observed. They were caused by significant energy contained in characteristic frequencies of the meshing and their harmonics. The most qualitatively significant increase in the amplitude is observed when the meshing frequency meets the resonant frequency of circa 0.9 kHz.

By comparing the time-frequency distributions obtained in these experiments and the frequency distributions obtained in modal analysis (Figure 7b,d), a conclusion can be drawn on the convergence of the results of resonant frequencies for the K_3 housing in the two research methods.

Analyzing the time-frequency distributions from the coasting test of the K_3 housing, we can see that this housing is characterized by the occurrence of resonant frequencies in the range of higher frequency bands, which also occurred for the Steel housing and did not occur for the K_1 and K_2 composite housings. However, after a comparison of qualitative changes in the distribution values for the Steel and K_3 housings, it can be noticed that the amplitude of vibrations and SPL of the K_3 housing in higher frequency bands is lower than that for the Steel housing. This indicates that the composite housing K_3 has a lower vibroactivity than the Steel housing also at higher rotational speeds.

### 3.2. Analysis of Vibration and Noise of Gearboxes Recorded at Constant and Variable Rotational Speeds and Different Loads of the Gearing 

In the subsequent phases of the research, the vibroactivity of steel and composite housings was evaluated at different rotational speeds and different loads of the gearing.

In experiments conducted in a circulating power FZG test stand—Figure 1—an assumption was made that measurements of vibration acceleration and acoustic pressure would be carried out:

- At a constant speed “*n*” of:
  ○ 600, 900, 1200, 1500, 1800, 2100 and 2400 rpm,
- At two unit loads at the gear meshing point “*Q*” of:
  ○ 1.5 and 0.75 MPa.

**Figure 8:**
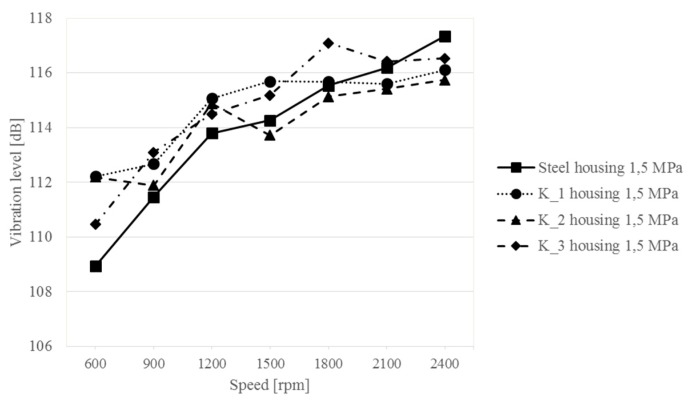
Changes in the vibration level of the upper gearbox plate at different rotational speeds of the gear—1.5 MPa load.

**Figure 9:**
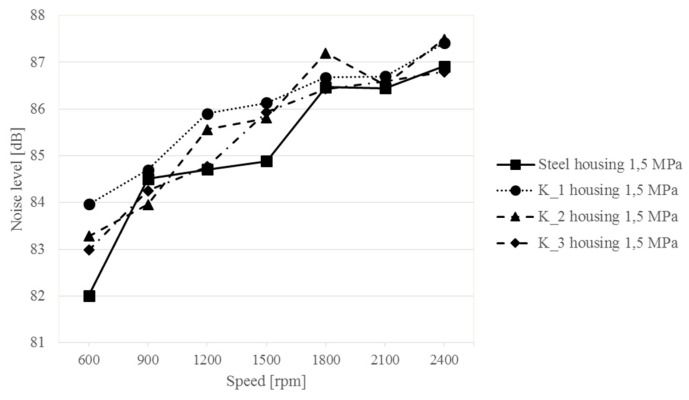
Changes in the sound level above the upper gearbox plate at different rotational speeds of the gear—1.5 MPa load.

**Figure 10:**
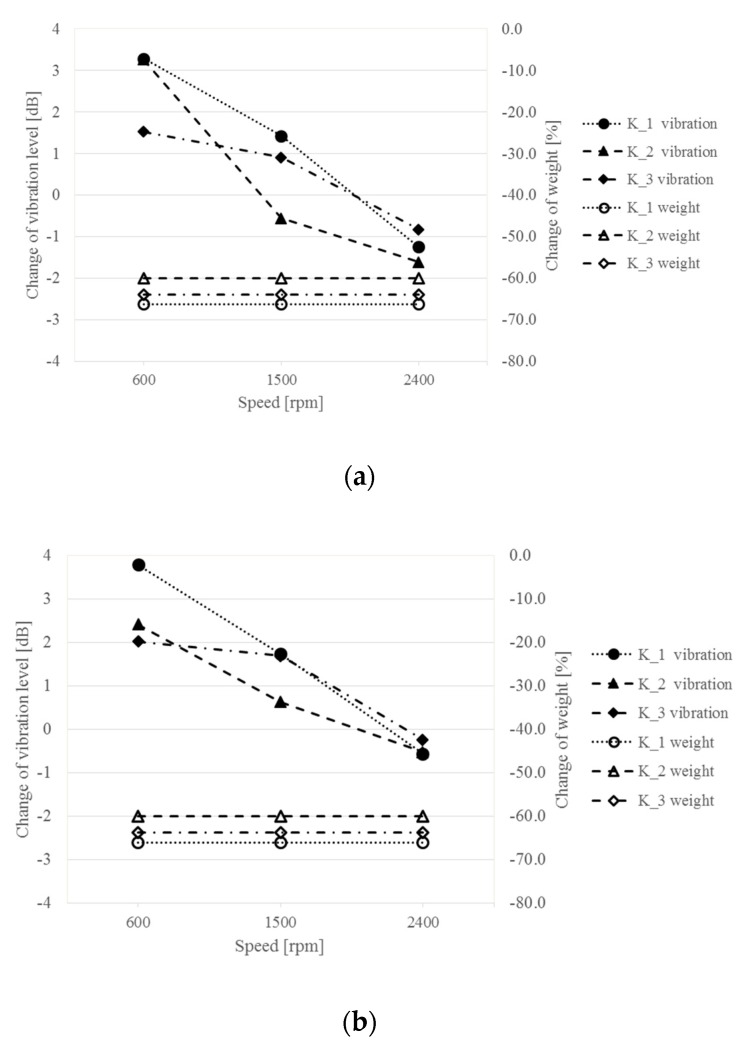
Change in the vibration level in (dB) and weight in (%)—measurement at the load: (**a**) 1.5 MPa; (**b**) 0.75 MPa.

**Figure 11:**
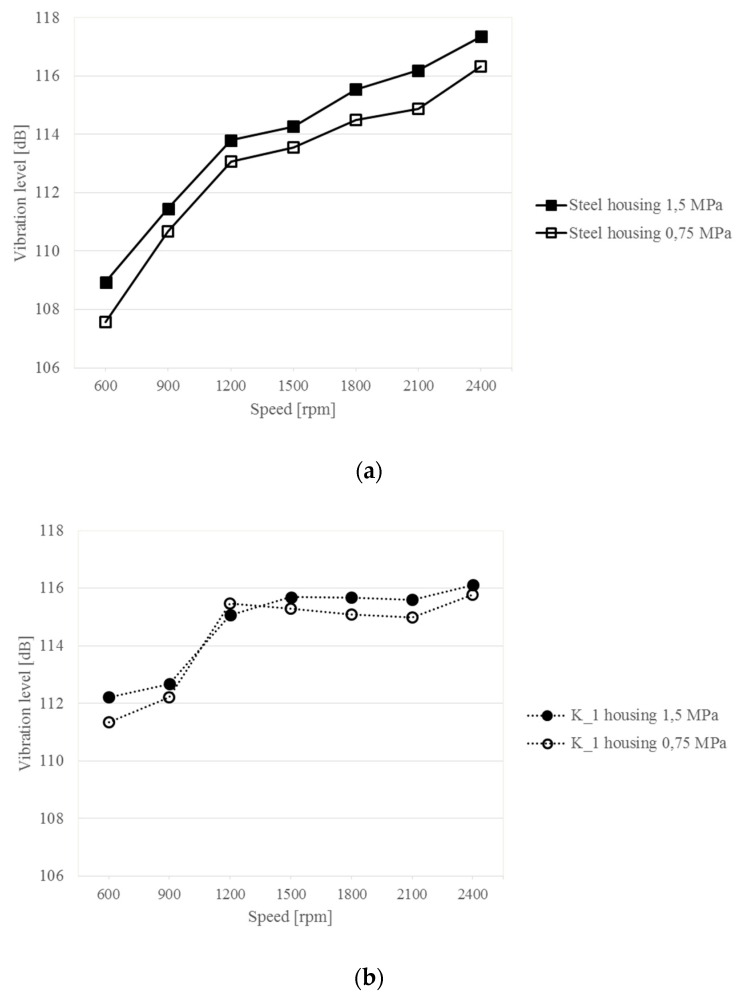

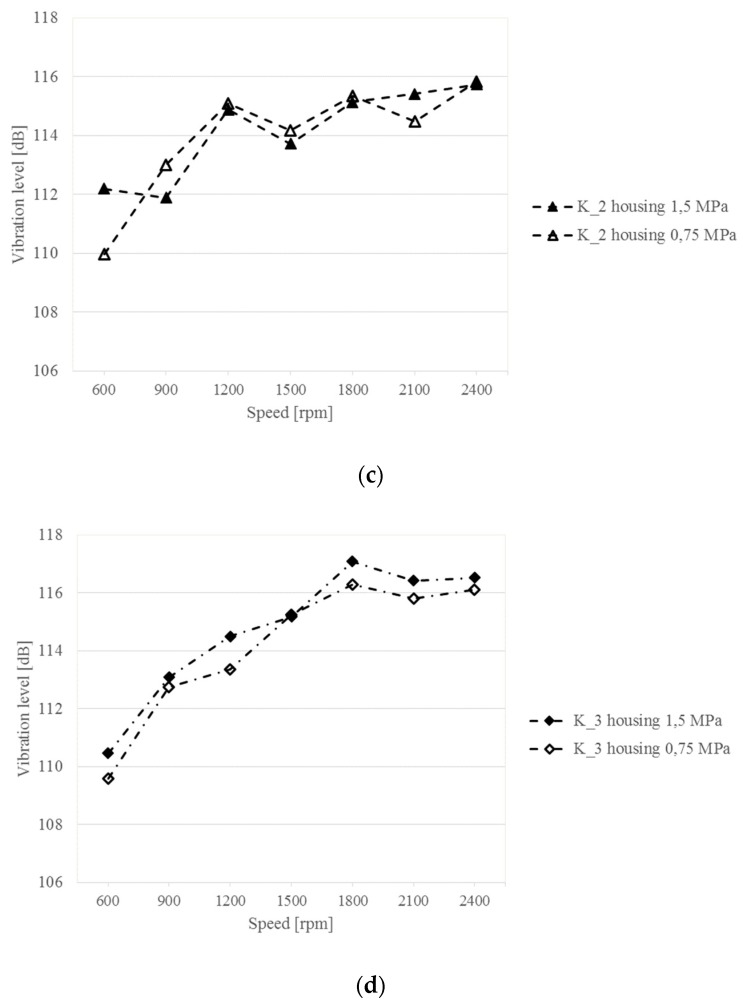
Changes in the vibration level of the upper gearbox plate at different rotational speeds of the gear and different loads.

Figure 8, Figure 9, Figure 10 and Figure 11 show the values of vibration and acoustic pressure levels averaged for five measurement points as a function of the rotational frequency of the wheel shaft (rotational speed of the wheel shaft). 

The vibration and acoustic pressure levels are presented in dB, with an assumption that the reference values were *a*_ref._ = 2 × 10^−5^ m/s^2^ and *p*_ref._ = 2 × 10^−5^ Pa, respectively.

Analyzing the results obtained, it can be stated that in the case of tests at a higher load applied to the gear, i.e., *Q* = 1.5 MPa, the differences in vibration generated by the upper gearbox housing change in two ways. Within the rotational speed range of 600–1200 rpm, the Steel housing has a vibration level lower by several decibels than the K_1–K_3 composite housings. The maximum vibration difference is 3 dB and decreases with an increasing rotational speed. From a rotational speed of 1500 rpm, it is observed in the results that the composite housings have lower vibration levels than Steel housing. This change occurs successively in the following order: for the K_2 housing at a speed of 1500 rpm, next at 2100 rpm for housing K_1, and at 2400 rpm for housing K_3. The examination also shows that a change of rotational speed in the range of 600–2400 rpm induces an increase in the vibration level of the Steel housing by 7.3 dB, whereas the changes are smaller for the composite housings and amount to 3.9 dB for the K_1 housing, 3.8 dB for K_2 and 6 dB for K_3. 

These results indicate that composite housings, with their much lower weight, are less sensitive to changes in rotational speed of the gear, and only the K_3 housing generates vibration levels that are the close to the Steel housing (Figure 4, Figure 5, Figure 6 and Figure 7). The results also confirm qualitative changes occurring in the time-frequency distributions, where an increase in the vibration amplitude of the steel housing was observed even at higher rotational speeds of the gear—interaction of characteristic meshing frequencies with modal frequencies of the gearbox housing. For K_1 and K_2 composite housings, no resonance is observed in higher frequency ranges, and therefore, no additional vibration excitation occurs at higher rotational speeds.

Measurements of acoustic pressure at a distance of 0.5 m from the upper plate of the housing showed that the use of different materials does not significantly affect the difference in the noise generated by the housings—differences below 2 dB (Figure 9). The differences are difficult to correlate. As the rotational speed increases, the sound level increases regardless of the housing tested. The change in sound levels in the analyzed range of rotational speed for the steel housing is 4.9 dB, and for the composite housings: 3.5 dB for K_1, 4.2 dB for K_2 and 3.8 dB for K_3. The tests also show that despite the use of much lighter composite materials for the gearbox housing structures, while maintaining geometric similarity, there was no clear increase in the noisiness of the gearbox.

Quantitative comparisons of the change in vibroactivity of composite housings K_1–K_3 in relation to the Steel housing were also made by determining the change in vibration level at three different gearbox rotational speeds: 600, 1500 and 2400 rpm. Figure 10 shows the obtained results of vibration level change measurements and the differences in weight of the composite housings used (in relation to the Steel housing). 

Analyzing the obtained results, it can be concluded that with an increase in the rotational speed of the gearbox, the composite housings selected for the study are characterized by a lower level of vibration than the Steel housing. Significant differences in the reduction of the vibration level were observed for a higher gear load of 1.5 MPa, while the K_2 housing showed the highest change in vibration of—1.6 dB with a weight reduction by 60%.

Further in this chapter, a study on the comparison of sensitivity of the Steel housings and the composite housings K_1–K_3 to a change in the load and rotational speed of the gearbox is presented. The obtained vibration levels of each of the tested housings were compared, depending on the gearbox rotational speed and two loads, 1.5 and 0.75 MPa. The results of these tests are shown in Figure 11.

The measurements and calculations allow affirming that the Steel housing is sensitive to changes in gear load regardless of the speed. As can be observed in Figure 11a, an increase in the gear load and rotational speed causes an increase in housing vibration in each case. Differences in the vibration level due to load variations are up to 1.5 dB.

Analyzing the test results obtained for the K_1 and K_2 composite housings, it can be noted that a change in the gear load affects the change in the vibration level in an ambiguous way. In some ranges of rotational speed, it can be observed that with increasing load the vibration level in higher, while in others it is lower. These results differ considerably from those obtained for the Steel housing.

The comparison of vibration also enables a quantitative and qualitative analysis of changes in the level of vibration of the composite housings with the increased rotational speed. It is noticeable here that the vibration level of the composite housings stabilizes after exceeding a given rotational speed and then increases only slightly. For the K_1 and K_2 housings, the stabilization takes place at 1200 rpm, and for K_3 at 1800 rpm. Relating the results of these tests of the composite housings to the results obtained for the steel housing, it can be concluded that the vibroactivity of the composite housings, with a much lower weight, stabilizes with an increasing rotational speed, which is not the case for the steel housing. Thus, the results of these tests confirm that the composite housings at a high rotational speed of the gear are characterized by lower vibroactivity and, at the same time, by a significantly lower mass than the steel housing.

## 4. Conclusions

The results presented in this paper show the changes in the vibroactivity of upper gearbox housings made of different materials, at different rotational speeds of the toothed gear and different loads of the gearing. The results from the research show that composite housings are less sensitive to changes in the rotational speed of the gear compared to the steel housing. The tests showed that at higher ranges of the rotational speed of the gear, the housings made of composite materials had a comparable or lower level of vibration. The tests and analyses of the vibroactivity of the housings performed at different loads of the gear allow the conclusion that the composite housings are less sensitive to changes in the load than the steel housing. Based on the tests performed and their results, it can be concluded that evaluation of the vibroactivity of gearbox housings made of different materials should be conducted in both the domain of time and frequency of the vibration signal and noise.

## Figures and Tables

**Table 1:** Geometrical parameters of gear wheels used in investigations.

Number of pinion teeth *z*_1_ (-)	38	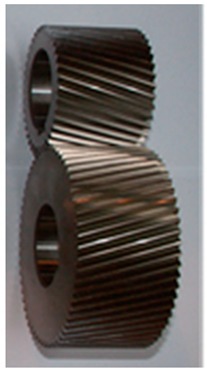
Number of wheel teeth *z*_2_ (-)	60	
Normal module *m_n_* (mm)	1.75	
Normal pressure angle *α*_on_ (°)	20	
Helix angle *β* (°)	15	
Distance between the centers of two gears *a_w_* (mm)	91.5	
Transverse contact ratio *ε_α_*	1.4	
Face contact ratio *ε_β_*	2.636	
Total contact ratio *ε_C_*	4	
Coefficient of pinion addendum modification *x*_1_	0.794	
Coefficient of wheel addendum modification *x*_2_	0.795	
Face width *b_w_* (mm)	56	
